# The Role of Personal Values in Forming Students’ Entrepreneurial Intentions in Developing Countries

**DOI:** 10.3389/fpsyg.2020.525844

**Published:** 2020-11-19

**Authors:** Saeid Karimi, Ahmad Salman Makreet

**Affiliations:** ^1^Department of Agricultural Extension and Education, Faculty of Agriculture, Bu-Ali Sina University, Hamedan, Iran; ^2^Master Student of Agricultural Innovation and Entrepreneurship, Department of Agricultural Extension and Education, Bu-Ali Sina University, Hamedan, Iran

**Keywords:** theory of planned behavior, entrepreneurial intentions, personal value, developing country, Iran, Afghanistan

## Abstract

The purpose of this study was to examine mechanisms through which personal values are associated with entrepreneurial intentions by integrating the theory of human values into the theory of planned behavior (TPB). Data were collected using a questionnaire from a sample of 452 agriculture students who were selected from two public universities in two Persian-speaking countries, namely Iran and Afghanistan. The results of structural equation modeling showed that individualistic personal values, that is, openness to change and self-enhancement values, are not directly related to entrepreneurial intentions. However, these personal values were indirectly related to entrepreneurial intentions through attitude toward entrepreneurship and perceived behavioral control. The results also showed no significant difference between the two countries in terms of the relationship between the personal values and three TPB anchors (i.e., attitude toward entrepreneurship, subjective norms, and perceived behavioral control) or the relationship between TPB anchors and entrepreneurial intentions. The study indicates how personal values play a role in explaining entrepreneurial intentions and establishes the utility of the TPB model in seeking a value–intention linkage in the field of entrepreneurship in developing countries. As a practical implication, the study suggests that educators of potential entrepreneurs should try to stimulate personal values more often because this fosters entrepreneurial intentions and their antecedents.

## Introduction

Entrepreneurship is one of the main drivers of innovation, productivity, job creation, and economic and social development (Carree and Thurik, 2003; van Stel et al., 2005; Karimi et al., 2014). Given the positive effects of entrepreneurship, understanding its determinants has become an important field of research. The entrepreneurship literature has shown that intentions play a key role in the decision to start a new business and are considered to be the most immediate and important variable in predicting entrepreneurs’ future behavior (Kolvereid and Isaksen, 2006; Adam and Fayolle, 2015; Kautonen et al., 2015). Therefore, understanding the antecedents of entrepreneurial intentions is crucial to the study of entrepreneurial behavior (Fitzsimmons and Douglas, 2011). Personal values represent potentially relevant variables in this respect (Morales et al., 2019). Individuals make important choices and decisions about their jobs and workplaces in accordance with their values, which are their “guiding principles in life” (Schwartz, 1992). These values are seen as a window through which potential actions and their desirability can be viewed (Schwartz, 1992; Holland and Shepherd, 2013). They affect a person’s attitude and behavior, and as guiding principles, they play a significant role in an individual’s decisions and actions (Schwartz, 2011). Although psychologists have previously examined the role of personal values in decision making and behavior (Rohan, 2000; Schwartz and Boehnke, 2004; Bardi et al., 2009; Morales and Holtschlag, 2013), relatively little research has focused on the way these values may enhance entrepreneurial intentions, particularly in the early stages of an entrepreneurial endeavor (Liñán et al., 2016; Gorgievski et al., 2018; Morales et al., 2019). Some studies have explored the relationship between entrepreneurial intentions and personal values in Western countries, but thus far, little research on this topic has been conducted in developing countries. To narrow this gap in the literature, the main purpose of this article was to examine the relationship between personal values and entrepreneurial intentions in two developing Persian-speaking countries, namely Iran and Afghanistan. It examines how individualistic personal values contribute to forming the intention to start a business in the context of emerging economies with their own culture, such as Iran and Afghanistan. To date, this relationship remains under-explored. It is also consistent with the call to reveal the role of personal values in entrepreneurship, particularly in developing countries (Fayolle and Liñán, 2014; Liñán and Fayolle, 2015; Liñán et al., 2016). The present study’s research model was developed by integrating Schwartz’s theory of personal values (Schwartz, 1992) into the well-known model of the theory of planned behavior (TPB; Ajzen, 1991). According to TPB, entrepreneurial intentions are developed from three antecedents, namely attitude toward entrepreneurship, subjective norms, and perceived behavioral control. The influence of personal values on these three antecedents was analyzed. It is a mediator model in which personal values are associated with entrepreneurial intentions through the three antecedents.

## Iranian and Afghan Contexts

Iran and Afghanistan are emerging economies of great strategic and economic importance in South Asia, each with a rich and ancient cultural heritage. These neighboring Persian-language countries have deep historical, religious, and cultural links. Iran is the second largest economy in the region and a member of the Next 11 owing to its high growth potential (O’Neill et al., 2005). A notable feature of Iran’s economy is its inclination toward entrepreneurship. Iran has implemented a series of economic policies that have generated a large number of knowledge-based entrepreneurial businesses (Levasseur et al., 2020). As stated by MacBride (2016), Iran has significant potential to become an entrepreneurial powerhouse due to its sizeable economy, the high level of education of its population, advantages in specific technologies, and its renewable infrastructure. Over the past decade, Afghanistan has also emerged as one of the most geo-strategically important countries in the South Asian region. After decades of political volatility, conflicts with the opposition and insecurity, Afghanistan’s economy has recently experienced unparalleled growth in GDP of 10–22% annually. Economic growth has been attributed to low GDP baselines, the denationalization of the economy, and importantly, the massive influx of international assistance (Azizi and Jamali, 2016).

The economic and political instability of Iran and Afghanistan might not appear to be a favorable condition for entrepreneurship. Paradoxically, turbulent sociopolitical environments and economies such as these may also offer opportunities for the development of an entrepreneurial culture—perhaps to an even greater extent than stable, developed economies (Karimi et al., 2017).

## Theoretical Framework

### Personal Values and the TPB

“Values are undoubtedly a construct that can help to explain behavior” (Cieciuch, 2017, p. 238). Individuals are stimulated to act in agreement with their values because they seek a sense of harmony between their beliefs and actions (Rokeach, 1973). Rokeach (1973) indicates that values are standards or criteria through which a person judges himself or others, and they influence his attitude and behavior. Schwartz (2011) believes that personal values are used as principles that guide the selection of decisions, attitudes, actions, and behavior. Therefore, values guide the person’s decision making, and they stimulate congruent behaviors (Bardi and Schwartz, 2003; Liñán et al., 2016). There are many theories about personal values; one of the most common and valid is Schwarz’s psychological theory of values. Schwartz’s theory of values is the best capital of social psychology, which describes a set of universal values by providing the concept and methodology (Maio, 2010; Schwartz, 2011). This theory explains the value system and the dynamic relations among the values. In his theory, Schwartz has expressed 10 types of personal values that have different motivational effects. According to Schwartz’s research, these 10 types of values exist in all cultures, and they have relatively similar meanings (Schwartz, 1994). Schwartz’s personal values include “power, achievement, hedonism, stimulation, self-direction, universalism, benevolence, tradition, conformity, and security.”

Schwartz (2006) suggests that the whole value structure could be grouped into the four value styles, including “self-transcendence, conservation, self-enhancement, and openness to change.” The first two value dimensions (i.e., self-transcendence and conservation) represent aspects of collectivism, and the second two value dimensions (i.e., self-enhancement and openness to change) represent aspects of individualism (Konsky et al., 2000). The dimension of openness to change includes the values of stimulation and self-direction. These values are related to things that could be motivational factors for individuals to pursue their mental interests and so to accept change. The dimension of self-enhancement includes the values of power and achievement. These values are related to social status, prestige, and personal success (Schwartz, 1992). The current study is focused on those individualistic values included in the self-enhancement and openness-to-change dimensions and mechanisms that link those values to entrepreneurial intentions. Individualistic values, such as openness to change and self-enhancement are particularly conducive to entrepreneurship and new venture creation (Noseleit, 2010; Schwartz, 2015; Gorgievski et al., 2018; Looi, 2019; Morales et al., 2019). Individuals who value openness to change, emphasizing independence and readiness for new ideas, actions, and experiences, and those who value self-enhancement, emphasizing pursuit of self-interests and dominance over others (Schwartz, 1992, 2011), are likely attracted to entrepreneurial career choices that offer the possibilities to fulfill those values. Entrepreneurial activity allows the realization of those values through offering high levels of autonomy and freedom and the possibility to lead others and obtain potentially high monetary returns (Gorgievski et al., 2018).

Entrepreneurial intentions refer to the intention of an individual to start a new business (Krueger, 2009). Forming entrepreneurial intentions is the first step in the long process of creating a new business (Kessler and Frank, 2009). Numerous models have been applied to clarify and explain entrepreneurial intentions; however, none of them have been as important, influential, and useful as the model of the TPB (Griffiths et al., 2009; Kautonen et al., 2015; Karimi et al., 2017; Karimi, 2019). Unlike the other models of entrepreneurial intentions, the TPB provides a robust and applicable theoretical framework. This model gives us the ability to understand and predict entrepreneurial intentions by considering both the individual and social factors (Liñán et al., 2016). According to this theory, three motivational factors, including perceived behavioral control (i.e., the individual’s perceived ease or difficulty in becoming an entrepreneur), attitude toward entrepreneurship (i.e., the degree to which the individual holds a positive or negative personal valuation about being an entrepreneur), and subjective norms (i.e., the individual’s perceived social pressure from family, friends, or significant others to carry out—or not to carry out—entrepreneurial behaviors) directly predict entrepreneurial intentions (Ajzen, 1991; Liñán et al., 2016). According to the TPB, other cognitive-level variables, such as personal values, should influence entrepreneurial intentions indirectly through their antecedents (Krueger, 2007). As explained in the introduction, the importance of personal values in shaping entrepreneurial intentions and their antecedents remains under-studied. Personal values are, therefore, incorporated into the research model so as to examine their effects on the three TPB antecedents and entrepreneurial intentions.

### Personal Values and Entrepreneurial Intentions

Individuals differ in their priorities for the values. Through implementing Schwartz’s theory in entrepreneurship, the structure of the values enables one to understand which values could stimulate entrepreneurial behaviors. As mentioned, “openness to change” and “self-enhancement” are considered as individualistic dimensions. According to Schwartz’s theory, when faced with a similar situation, people make different decisions, depending on their value priorities, and then take action (Schwartz, 2006). Thus, people who prefer self-direction or stimulation values are more likely to be attracted to challenging jobs, and those who give priority to the safety value may find the same job unattractive or threatening (Jaén and Liñán, 2013). People who emphasize “openness to change,” place great importance on independent thinking and action. They seek adventure, stimulation, variety, novelty, and new ideas and experiences. Their emphasis on “self-enhancement” increases their satisfaction with success and personal gain even at the expense of the loss of others. Gaining dominance or control over people and resources and having social recognition and power are valuable to these people, and all of these values are related to entrepreneurial activity (Holland and Shepherd, 2013; Tomczyk et al., 2013; Liñán et al., 2016). In the field of business, people who are open to change look for new ways to accomplish goals and use their intellectual capacity creatively to develop new goods and services (Holland and Shepherd, 2013). Knowing that individuals choose occupational and career opportunities that are commensurate with their values (Schwartz, 1992), it can be said that those with a greater sense of openness to change are more likely to become entrepreneurs because they prioritize autonomy and curiosity, are fearless, and can tolerate change (Morales et al., 2019).

Self-enhancement is associated with individual rewards and a sense of progress. Ambitious and success-oriented people seek social power and credibility (Holland and Shepherd, 2013). Therefore, people with self-enhancing values are more likely to form high entrepreneurial intentions (Morales et al., 2019).

Although very little research has been done on personal values of entrepreneurs, however, even these few studies indicate the relationship between individualistic values and entrepreneurial intentions and behavior (Espıritu-Olmos and Sastre-Castillo, 2015; Liñán et al., 2016; Looi, 2019). In their study, Moriano et al. (2007) report that individualistic values (such as power, development, stimulation, and self-centeredness) are positively related to entrepreneurial intentions of Hispanic students. The results of Liñán et al. (2016) show that individualistic values, i.e., openness to change and self-enhancement, have a positive relationship with entrepreneurial intentions. Also, the study on four European countries (Germany, Netherlands, Spain, and Poland) shows that openness to change and self-enhancement are positively related to entrepreneurial intentions (Gorgievski et al., 2018). Finally, a study by Morales et al. (2019) in 28 European countries shows that individuals with higher levels of individualistic values, openness to change, and self-enhancement are more likely to become entrepreneurs. In this regard, Liñán et al. (2016) states that, because entrepreneurship is a complex process with multiple stages and steps, each individualistic feature may be activated during one of these stages. That is, preferring openness to change can be related to the identification and evaluation of opportunities, and the emphasis on self-enhancement can be related to the exploitation of opportunities. Thus, individuals who value both groups of individualistic values (openness to change and self-enhancement) are much more motivated to perform behaviors required at each stage of the entrepreneurial process (Liñán et al., 2016). One could expect these people to have more entrepreneurial intentions. Therefore, the following hypotheses are proposed:

H1. Openness to change is positively related to entrepreneurial intentions.

H2. Self-enhancement is positively related to entrepreneurial intentions.

### Personal Values and the TPB Components

Scholars argue that personal values, in addition to their direct effects, may also have indirect effects on intentions and behavior via different cognitive processes (Bardi and Schwartz, 2003; Morales et al., 2019). Due to this, they suggest exploring the role of mediating variables in the relationship between personal values and entrepreneurial intentions (Morales et al., 2019). Also, according to the TPB, values are background factors that could affect intentions and behaviors indirectly by guiding a person’s beliefs and attitudes (Ajzen and Fishbein, 2005). In addition, based on the value-attitude-behavior hierarchy theory (Homer and Kahle, 1988), personal values influence behavior indirectly through attitudes. In other words, values play an essential role in forming attitudes that lead to particular behaviors. Thus, it is expected that personal values that are compatible with entrepreneurship, that is, self-enhancement and openness to change, could have an indirect effect on entrepreneurial intentions through attitude toward entrepreneurship and perceived behavioral control. The study by Kruse et al. (2019) supports these hypotheses. The study of Liñán and Kurczewska (2017) also suggests the impact of individual values on the readiness to identify entrepreneurial opportunities, which itself could lead to improved control of the perceived entrepreneurial behavior. The study conducted by Karimi et al. (2019) reports that individualistic values are positively related to attitude toward entrepreneurship and perceived behavioral control. Experts believe that values compatible with entrepreneurship stimulate entrepreneurial skill acquisition. This could lead to improved confidence in individuals’ entrepreneurial capabilities and abilities, and thus, their entrepreneurial self-efficacy or their perceived behavioral control is enhanced. Also, in line with the empirical research findings, they argue that personal values congruent with entrepreneurship prepare individuals to develop a particular and positive attitude toward entrepreneurship as a desirable profession (Gorgievski et al., 2018). According to Homer and Kahle (1988), personal values may shape attitudes by guiding individuals to look for objects that will satisfy their values and, thus, to consider those objects more desirable and interesting. For instance, individualistic values foster the pursuit of self-direction and power (Schwartz, 1992). In this sense, individuals with high individualistic value orientations would be likely to view a favorable attitude toward entrepreneurship as a sign of self-direction and power. For example, in a study with a sample of four European countries, the indirect effects of the values of openness to change and self-enhancement through attitude toward entrepreneurship and perceived behavioral control are confirmed (Gorgievski et al., 2018). Thus, the following hypotheses are derived:

H3. Openness to change is positively related to attitude toward entrepreneurship.

H4. Self-enhancement is positively related to attitude toward entrepreneurship.

H5. Openness to change is positively related to perceived behavioral control.

H6. Self-enhancement is positively related to perceived behavioral control.

Openness to change and self-enhancement may reduce people’s readiness to follow the expectations of people they care about (such as family members and friends). The values of openness to change emphasize independence in thought and action, and they encourage risky behavior and innovation (Schwartz, 1992). They may make the wishes and desires of others less important to the person. Self-enhancement values focus more on the individual and his interests, and thus, they can reduce one’s willingness to consider others’ views and opinions (Bernard et al., 2003). Therefore, individuals with higher individualistic values tend to focus more on their own abilities, characteristics, and goals than on the thoughts, feelings, and actions of others. In other words, they are thought to be less concerned about meeting the expectations of others and social norms. As a result, they are liable to display relatively lower levels of subjective norms and are also less likely to seek to conform to the opinions of others when starting their own businesses (Karimi et al., 2019). A recent study indicates that self-enhancement and openness to change values are negatively related to subjective norms (Gorgievski et al., 2018). Therefore, the following is hypothesized:

H7. Openness to change is related negatively to subjective norms.

H8. Self-enhancement is related negatively to subjective norms.

## Materials and Methods

### Participants

The present survey was conducted on undergraduate junior and senior agricultural students in two Universities of Iran and Afghanistan. These students were targeted because they have a relatively more clear vision of their future career plans and decisions than freshmen, and they are more likely involved in their professional decisions as well (Karimi et al., 2014).

### Procedure

The questionnaire was originally developed in English and then carefully translated into Persian by the research team using the translation-back-translation technique (Hambleton, 1994) to ensure that item meaning was preserved through the translation process. With the permission of the lecturers, the questionnaires were distributed for voluntary completion by students at the beginning of a class session during the 2019–2020 academic year. Before filling out the questionnaire, students were asked to read a cover letter that explained the general purpose of the study and that ensured confidentially of individual responses. The students were given 20 min to complete the questionnaire and received a small gift for doing so.

The response rate was 78% with a total of 600 questionnaires distributed and 470 questionnaires collected. The completed questionnaires were screened for missing data and outliers, which resulted in 452 usable questionnaires (Iran: 231, Afghanistan: 221). Overall, 53.7% of the respondents were female, and the rest (46.3%) were male; their mean age was 23.22 and about 67.5% of them had attended the entrepreneurship classes or courses. Less than one third of the students had entrepreneurial experience (30.1%). Also, more than half of them (52.1%) were familiar at least with one entrepreneur and his business. Table 1 shows the sample characteristics in each country.

**TABLE 1:** Description of the respondents’ characteristics.

	Afghanistan (*n* = 221)		Iran (*n* = 231)		Total sample (*N* = 452)	
	Mean	SD	Mean	SD	Mean	SD
Age (years)	23.19	4.30	23.94	3.63	23.49	5.03
Gender (0 = female, 1 = male)	0.54	0.49	0.18	0.38	0.76	0.43
Entrepreneurship education (1 = yes, 0 = no)	0.33	0.47	0.22	0.42	0.44	0.53
Entrepreneurship experience (1 = yes, 0 = no)	0.27	0.44	0.23	0.42	0.31	0.46
Knowing entrepreneur (1 = yes, 0 = no)	0.52	0.50	0.37	0.48	0.69	0.47

*SD, standard deviation.*

### Measurement Instrument

Schwartz’s Portrait Value Questionnaire (Schwartz et al., 2001; Schwartz, 2006) was used to measure personal values. The questionnaire consists of 40 items with a six-point Likert scale (ranging from 1 to 6) that describes some people and asks the respondent how much each person is similar or dissimilar to himself. The values of openness to change (including self-direction and stimulation) were measured using seven items. Also, self-enhancement values (including achievement and power) were measured using seven items. Cronbach’s alpha coefficient for “openness to change” and “self-enhancement” were 0.73 and 0.70, respectively.

The Entrepreneurial Intention Questionnaire (Liñán and Chen, 2009) was used to measure the variables of the TPB. This questionnaire measures the four variables of the TPB using a 5-point scale. The entrepreneurial intentions scale consists of six items with Cronbach’s alpha coefficient of 0.83. Attitudes toward entrepreneurship were measured through five items with a Cronbach’s alpha coefficient of 0.82. Three items were used to measure subjective norms with a Cronbach’s alpha coefficient of 0.81. The perceived behavioral control scale was also measured using six items that were a combination of self-efficacy and controllability. The Cronbach’s alpha coefficient for this scale was 0.83. Finally, age (years) and gender (0 = female, 1 = male) were included as control variables. These are typical examples of demographic variables affecting entrepreneurship, particularly in student samples (Karimi et al., 2014; Shirokova et al., 2016; Liñán et al., 2016)

### Statistical Analysis

The partial least squares approach to structural equation modeling (PLS-SEM) was used to analyze the research model and the relationships among the variables. One of the major advantages of PLS-SEM is that it helps to predict the target variables (entrepreneurial intentions in the present study) and to measure the predictive power of its antecedents. The PLS-SEM analysis is done in two steps. The first is the assessment of reliability and validity of the measurement model. Once the measurement model’s adequacy is established, the second step deals with the assessment of the structural model by specifying the causal relationships in accordance with the hypotheses (Hair et al., 2017). The data was analyzed using Smart PLS 3.2.8 software (Ringle et al., 2017), which is one of the most commonly used SEM software.

### Results

The mean, standard deviation, and correlation coefficients among the variables are shown in Table 2. As was mentioned before, the scale used to measure the variables was the Likert 5-point scale, and in this scale, 3 is considered as the neutral value. The values below this neutral value represent the negative values of the scale, and those values higher than that represent the positive values of the related scale. As shown in Table 2, the mean score of entrepreneurial intentions is 3.43, which falls slightly above the neutral value of 3. This indicates that the students were moderately inclined to start a new business. Also, the mean score of the other variables was slightly above average.

**TABLE 2:** Means, standard deviations (SD), correlations, and square roots of average variance extracted estimates (in bold) for all variables for the total sample (*N* = 452).

Variables	Mean	SD	1	2	3	4	5	6	7	8
1. Age	23.19	4.30	–							
2. Gender	0.54	0.49	0.20**	–						
3. Entrepreneurial intentions	3.43	1.03	–0.04	−0.20**	**(0.77)**					
4. Attitude toward entrepreneurship	3.79	0.97	–0.04	−0.16**	0.75**	**(0.76)**				
5. Subjective norms	3.69	1.10	–0.01	–0.08	0.60**	0.67**	**(0.85)**			
6. Perceived behavioral control	3.11	0.99	0.06	−0.24**	0.73**	0.66**	0.54**	**(0.74)**		
7. Openness to change	4.87	0.82	–0.02	0.02	0.45**	0.50**	0.29**	0.42**	**(0.65)**	
8. Self-enhancement	4.64	0.84	0.04	–0.07	0.44**	0.35**	0.29**	0.34**	0.42**	**(0.66)**

**p < 0.01.

### Assessment of the Measurement Model

The first step in analyzing the measurement model in PLS involves examining the goodness of fit of the global model using the SRMR index. The SRMR value of the measurement model of this study was 0.07, which was less than the suggested value of 0.08; thus, the model’s goodness of fit was verified. After the verification of the model’s fit, the assessment of the measurement model focused on the analysis of the validity and reliability of the variables. To assess the reliability of the model, the combined reliability and Cronbach’s alpha were computed. The results are shown in Table 4. As is shown, the results verified the reliability of the variables because they were all above the proposed value of 0.7. The convergent validity of the variables was assessed by the average variance extracted (AVE) index. As shown in Table 4, the AVE coefficient for all the variables was close to or greater than 0.5, indicating a good convergent validity of the measurement model.

Finally, the discriminant validity of the constructs was tested using the Fornell and Larcker (1981) method. According to Table 2, the square root of AVE for all of the constructs (the bold values within the parentheses on the table’s diagonal) is greater than the correlation value among that construct and the other constructs, indicating good discriminant validity of the measurement model. The assessment of the measurement model proves that all the constructs have appropriate reliability and validity.

### Assessment of the Structural Model

After verifying the validity and reliability of the measurement model, the next step was to evaluate the results of the structural model in order to examine the significance of the research hypotheses and the percentage of the predicted variance. The *R*^2^ values shown in Table 3 represent the explained variance of each latent variable. As is shown, the model can explain 77, 30, 27, and 17% of the variance of entrepreneurial intentions, attitude toward entrepreneurship, perceived behavioral control, and subjective norms, respectively. Moreover, the results of the Aston-Geyser test prove that the model has a good prediction power because the *Q*^2^ values are positive for all the latent variables.

**TABLE 3:** Assessment results of the measurement and structural models.

Variable	Measurement model			Structural model	
	α	CR	AVE	*Q*^2^	*R*^2^
Self-enhancement	0.70	0.78	0.43	–	–
Openness to change	0.73	0.81	0.42	–	–
Subjective norms	0.81	0.89	0.72	0.16	0.17
Attitude toward entrepreneurship	0.82	0.87	0.58	0.27	0.30
Perceived behavioral control	0.83	0.88	0.55	0.21	0.27
Entrepreneurial intentions	0.84	0.88	0.56	0.77	0.77

*α: Cronbach’s alpha; AVE: average variance extracted; CR: composite reliability; O^2^: predictive relevance; R^2^: coefficient of determination*

To test the hypotheses, the standardized path coefficients (β) and the effect size (*f*^2^) of the assumed relationships were analyzed. As is shown in Table 4, the results of bootstrapping analysis indicate that the relationship between attitude toward entrepreneurship (β = 0.34; *p* < 0.01), subjective norms (β = 0.07; *p* < 0.05), perceived behavioral control (β = 0.56; *p* < 0.01), and entrepreneurial intentions is significant. The effect size is moderate for attitude toward entrepreneurship–entrepreneurial intentions (*f*^2^ = 0.19) and large for perceived behavioral control–entrepreneurial intentions (*f*^2^ = 0.69). Although the effect of subjective norms on entrepreneurial intentions is significant, the effect size is less than the 0.02 threshold for the small effects (*f*^2^ = 0.01). The direct relationship between openness to change (H1: β = −0.02; *p* > 0.05) and self-enhancement (H2: β = 0.05; *p* > 0.05) with entrepreneurial intentions is not significant, so H1 and H2 are not supported.

**TABLE 4:** Direct, indirect, and total effects of the research model for the total sample (*N* = 452).

Hypotheses	Relation			β	CI	*f*^2^	Supported
	**Control Variables**						
	Age	→	Entrepreneurial intentions	−0.08*			
	Age	→	Attitude toward entrepreneurship	−0.08*			
	Age	→	Subjective norms	–0.03			
	Age	→	Perceived behavioral control	0.01			
	Gender	→	Entrepreneurial intentions	–0.02			
	Gender	→	Attitude toward entrepreneurship	−0.17**			
	Gender	→	Subjective norms	–0.08			
	Gender	→	Perceived behavioral control	−0.23**			
	**Direct effects**						
	Attitude toward entrepreneurship	→	Entrepreneurial intentions	0.34**	0.26–0.42	0.19	Yes
	Subjective norms	→	Entrepreneurial intentions	0.07	0.01–0.13	0.01	No
	Perceived behavioral control	→	Entrepreneurial intentions	0.56**	0.48–0.62	0.69	Yes
H1	Openness to change	→	Entrepreneurial intentions	–0.02	−0.07 to 0.05	0.00	No
H2	Self-enhancement	→	Entrepreneurial intentions	0.05	−0.01 to 0.10	0.01	No
H3	Openness to change	→	Attitude toward entrepreneurship	0.41**	0.31–0.52	0.13	Yes
H4	Self-enhancement	→	Attitude toward entrepreneurship	0.15**	0.04–0.24	0.02	Yes
H5	Openness to change	→	Perceived behavioral control	0.39**	0.29–0.50	0.11	Yes
H6	Self-enhancement	→	Perceived behavioral control	0.09	−0.01 to 0.23	0.00	No
H7	Openness to change	→	Subjective norms	0.30**	0.18–0.42	0.06	No
H8	Self-enhancement	→	Subjective norms	0.14**	0.01–0.25	0.01	No
	**Indirect effects**						
	Openness to change → attitude toward entrepreneurship	→	Entrepreneurial intentions	0.14**	0.10–0.19		Yes
	Openness to change → subjective norms	→	Entrepreneurial intentions	0.02	0.00–0.04		No
	Openness to change → perceived behavioral control	→	Entrepreneurial intentions	0.22**	0.15–0.21		Yes
	Self-enhancement → attitude toward entrepreneurship	→	Entrepreneurial intentions	0.05*	0.01–0.09		Yes
	Self-enhancement → subjective norms	→	Entrepreneurial intentions	0.01	−0.00 to 0.02		No
	Self-enhancement → perceived behavioral control	→	Entrepreneurial intentions	0.05	−0.02 to 0.11		No
	**Total effects**						
	Openness to change	→	Entrepreneurial intentions	0.37**	0.25–0.45		
	Self-enhancement	→	Entrepreneurial intentions	0.11**	0.06–0.30		

*p < 0.05; **p < 0.01; β, standardized path coefficient; CI, confidence interval; *f*^2^, effect size; VAF, variance accounted for; N/A, not applicable.

The findings indicate that there are positive and significant relationships of openness to change (H3: β = 0.41; *p* < 0.01) and self-enhancement (H4: β = 0.15; *p* < 0.01) with attitude toward entrepreneurship. Hypotheses 3 and 4 are, thus, supported. In addition, openness to change shows a positive and significant association to perceived behavioral control (H5: β = 0.39; *p* < 0.01). Hypothesis 5 is also, thus, supported, and no significant relationship of self-enhancement with perceived behavioral control is found (H6: β = 0.09; *p* > 0.05). The effect sizes are small for openness to change–attitude toward entrepreneurship (*f*^2^ = 0.13), self-enhancement–attitude toward entrepreneurship (*f*^2^ = 0.02), and openness to change–perceived behavioral control (*f*^2^ = 0.11). However, openness to change (H7: β = 0.30; *p* < 0.01) and self-enhancement (H8: β = 0.14; *p* < 0.01) are positively related to subjective norms, which is opposed to the proposed directions. Consequently, H7 and H8 are, thus, not supported.

Regarding the control variables, a higher age was found to have a negative effect on entrepreneurial intentions (β = −0.08; *p* < 0.05) and attitude toward entrepreneurship (β = −0.08; *p* < 0.05). Furthermore, females had higher attitude toward entrepreneurship (β = −0.17; *p* < 0.01) and perceived behavioral control (β = −0.23; *p* < 0.01).

To test the mediation effects, we followed the suggestions of Preacher and Hayes (2004, 2008) by bootstrapping the indirect effects method (bootstraps = 5000). The bootstrapping method is the most powerful and appropriate non-parametric technique to test the mediation effect in PLS-SEM (Preacher and Hayes, 2008). As shown in Table 4, the indirect effect of openness to change on entrepreneurial intentions via attitude toward entrepreneurship [β = 0.14, *t* = 5.547; *p* < 0.01, 95% CI = (0.10, 0.19), VAF = 87%] and perceived behavioral control [β = 0.22, *t* = 6.521; *p* < 0.01, 95% CI = (0.15, 0.21), VAF = 91%] was significant. Furthermore, the lower- and upper-level confidence intervals did not straddle the value of zero, supporting the existence of mediation effects (Preacher and Hayes, 2008). According to the results, the indirect path from self-enhancement to entrepreneurial intentions via attitude toward entrepreneurship is significant (β = 0.12) with a 95% CI excluding zero (0.01–0.09). However, the other mediation effects of perceived behavioral control and subjective norms were not significant (Table 4).

The total effects also show that the three main predictors of entrepreneurial intentions are perceived behavioral control, openness to change, and attitude toward entrepreneurship, and subjective norms are the weakest predictor of entrepreneurial intentions (Table 4).

### Multigroup Analysis

**FIGURE 1:**
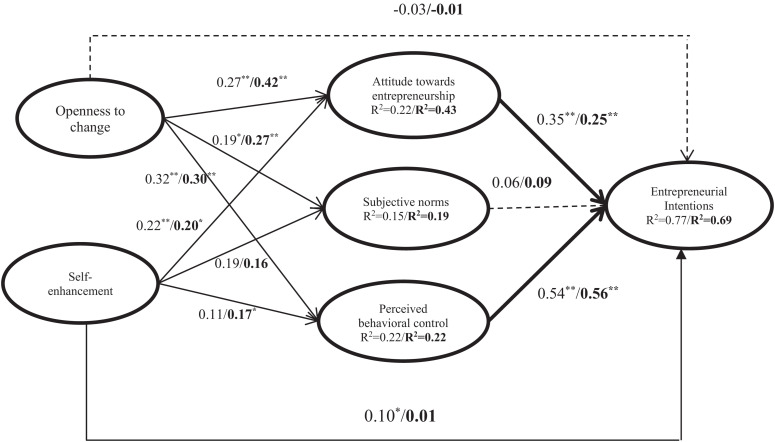
Structural model results for the Iranian (*n* = 231) and Afghan (n = 221) samples. Iran/**Afghanistan**; ^∗^*p* < 0.05, ^∗∗^*p* < 0.01; SRMR = 0.07/**0.08**; relations are controlled for age and gender.

In order to analyze the differences in the causal relationships of the model between Iran (*n* = 231) and Afghanistan (*n* = 221), a multigroup PLS-GMA analysis was performed (Hair et al., 2014). For both models, the SRMR value was less than 0.09, indicating a good fit (Figure 1). The results obtained from the bootstrapping procedure with 5000 resamples showed that there is no significant difference between Iran and Afghanistan on the assumed relationships in the research model (Table 5).

**TABLE 5:** Multigroup analysis of the path coefficients between the Iranian (*n* = 231) and Afghan (*n* = 221) samples.

Relation			β (Iran)	β (Afghanistan)	Path coefficients difference	*p-*Value
Attitude toward entrepreneurship	→	Entrepreneurial intentions	0.35	0.25	0.10	0.23
Subjective norms	→	Entrepreneurial intentions	0.06	0.09	0.03	0.70
Perceived behavioral control	→	Entrepreneurial intentions	0.54	0.56	0.02	0.75
Openness to change	→	Entrepreneurial intentions	−0.03	−0.01	0.02	0.72
Self-enhancement	→	Entrepreneurial intentions	0.10	0.01	0.09	0.19
Openness to change	→	Attitude toward entrepreneurship	0.27	0.42	0.15	0.22
Openness to change	→	Subjective norms	0.19	0.27	0.08	0.56
Openness to change	→	Perceived behavioral control	0.32	0.30	0.02	0.82
Self-enhancement	→	Attitude toward entrepreneurship	0.22	0.20	0.02	0.65
Self-enhancement	→	Subjective norms	0.19	0.16	0.03	0.65
Self-enhancement	→	Perceived behavioral control	0.11	0.17	0.06	0.70

## Discussion

Drawing upon the TPB and the theory of basic human values as well as previous research on entrepreneurial intentions, the purpose of the present study was to investigate the role of the two personal values, i.e., “openness to change” and “self-enhancement” in forming students’ entrepreneurial intentions. In this study, using a structural equation model, a mediation model was studied on a sample of 452 agriculture students in the two countries of Iran and Afghanistan.

The results show that the personal values are not directly related to entrepreneurial intentions; instead they have an indirect relationship with it through its antecedents in the TPB in the developing country context. Previous studies have also found that the motivational factors in the model of planned behavior mediate the relationship between students’ personal values and entrepreneurial intentions (Liñán et al., 2016; Gorgievski et al., 2018). These results support the basic assumption of the TPB. According to this assumption, the individual and personality variables, such as personal values, have an indirect effect on entrepreneurial intentions and motivational factors, such as attitudes, have a mediating role in such a relationship (Fishbein and Ajzen, 2011).

Specifically, in the relationship between individualistic personal values, perceived behavioral control, and attitudes toward entrepreneurship, the expected positive effects were found, confirming hypotheses 2 and 3. These results are in line with previous research that suggests openness to change and self-enhancement lead to a more favorable evaluation (attitude toward entrepreneurship) and more perceived ability and control (perceived behavioral control) regarding the process of new business creation (Gorgievski et al., 2018). In contrast to our hypotheses, it was found that individualistic personal values are positively related to subjective norms, which is not consistent with previous findings (Gorgievski et al., 2018; Karimi et al., 2019). It appears that, for Iranian and Afghan students, openness to change and self-enhancement may be positive predictors of subjective norms. One plausible explanation for this finding might be related to cultural contexts. Further research is needed to clarify this issue. In addition, there was no significant relationship between subjective norms and entrepreneurial intentions. It may be argued that part of these results may be a consequence of our operationalization of the subjective norms construct. We return to this in the limitations section.

The results also show that individualistic values are important for entrepreneurial careers and the selection of future jobs (Sagiv, 2002; Knafo and Sagiv, 2004). Therefore, they should be taken into account when studying the determinants of entrepreneurial intentions. The individualistic values are related to entrepreneurship; however, a particular dimension of the individualistic values (i.e., openness to change) further stimulates the entrepreneurial intentions of the undergraduate agriculture students. In other words, the psychological motivations, i.e., self-direction and stimulation (such as excitement, novelty, independence, and autonomy) motivate the entrepreneurial intentions of Iranian and Afghan undergraduate students more than economic motivations (i.e., the progress and power) although this is an indirect relation. Although Liñán et al. (2016) report that the values of self-enhancement in Spain are more related to entrepreneurial intentions than openness to change; this difference in Western and Eastern countries requires further investigation. The results show that perceived behavioral control and, to a lesser extent, attitude toward entrepreneurship mediates the effects of the individualistic values on entrepreneurial intentions. Thus, the research literature extends the relationship between personal values and entrepreneurial career choices (Liñán et al., 2016; Gorgievski et al., 2018).

The results of the present study indicate that the relative importance of the motivational factors in the TPB model for agriculture students of Iran and Afghanistan is such that perceived behavioral control has the strongest and subjective norms has the weakest relationship with their entrepreneurial intentions. This result suggests that the formation of students’ entrepreneurial intentions is based more on personal considerations than normative and social considerations. In other words, decision making about a future career may be of great importance to the person and one pays less attention to the opinions of others in this regard. Another possible explanation concerns the Iranian and Afghan economic contexts. The high rate of youth unemployment in Iran and Afghanistan could well limit the effects of perceived approval or disapproval of entrepreneurial activity on the entrepreneurial intentions of students. Start-ups are mostly driven by necessity in these countries (Muhammad et al., 2011; Karimi et al., 2017). It is, thus, possible that the effect of the opinions of others is limited due to the necessity of doing something and, thus, starting a new business. Given unstable economic and political country conditions, moreover, confidence in one’s ability to start and run a business can be expected to be a strong predictor of entrepreneurial intentions (Karimi et al., 2019). This result is in agreement with the results of the previous studies in Iran (Karimi et al., 2014, 2017).

The antecedents of entrepreneurial intentions are often considered as factors of culture, context-specific and dependent on the environment (Lüthje and Franke, 2003; Karimi et al., 2017). The factors in the TPB model have different levels of impact across different countries (Engle et al., 2010; Moriano et al., 2012). Therefore, it was expected that the relationships between personal values, the TPB’s antecedents, and entrepreneurial intentions would be different between Iran and Afghanistan. However, the results show that the relationship between personal values (i.e., openness to change and self-enhancement) and the variables of the TPB (i.e., attitude toward entrepreneurship, subjective norms, perceived behavioral control, and entrepreneurial intentions) and the relationship between the antecedents of entrepreneurial intentions and entrepreneurial intentions are not different among students of the two countries. Although this result requires further research, one possible reason may be that young people of Generation Y share common perceptions, values, and attitudes (Charters et al., 2011), and globalization has had more impact on the students than the contextual variables of each country.

### Implications

From a theoretical perspective, the results of this study contribute to the development of the literature on entrepreneurial intentions, and in particular, they emphasize the role of personal values as an intrinsic factor that is neglected in the entrepreneurship literature in forming entrepreneurial intentions (Liñán et al., 2016; Gorgievski et al., 2018; Kruse et al., 2019). The present study establishes the utility of the application of the TPB model in seeking values–entrepreneurial intentions linkage. It shows that Schwartz’s (1992,^1994^) and Ajzen’s (1991) theoretical frameworks are highly compatible in predicting entrepreneurial intentions, confirming previous studies that have examined this integration (Liñán et al., 2016; Morales et al., 2019). The results also respond to the recent calls for the study of the role of personal values in entrepreneurship, especially in developing countries (Fayolle and Liñán, 2014; Liñán and Fayolle, 2015; Liñán et al., 2016). Moreover, our results support the assertion that external variables, such as personal values, may indirectly influence entrepreneurial intentions via the antecedents described in the TPB (Fishbein and Ajzen, 2011).

From a practical perspective, the results of this study could help educators and policymakers in the promotion of entrepreneurship (Goodnow, 1997). Because values are deeply rooted in an individual’s life, and they are formed at the early stages of life, they can be incorporated as part of a school’s curriculum in a country’s long-term policy to encourage progression, independent thinking, creativity, and problem solving and, hence, entrepreneurial activity (Morales et al., 2019). The individualistic values are useful tools for pupils and university students to decide whether to pursue entrepreneurship education and the related disciplines for a future career in entrepreneurship or not. To enhance students’ entrepreneurial intentions through educational interventions, universities could identify individuals with high levels of openness to change and prepare them to become potential entrepreneurs (Gorgievski et al., 2018). Educators and career counselors should facilitate the formation of individualistic values among students and graduates. For example, using entrepreneurial role models that improve openness to change, they could help students to be prepared for more challenging and highly demanding careers, such as entrepreneurship. Educators and career counselors could also consider how the values relate to the components of the TPB in forming entrepreneurial intentions. To enhance entrepreneurial intentions, educators and counselors could improve perceived behavioral control and positive attitude toward entrepreneurship. Using entrepreneurial role models, conducting case studies on successful entrepreneurs, and playing the films and stories of their lives in the classroom could help to increase the motivational factors. Also, launching small businesses for students along with internship in entrepreneurial companies will expose students to the real world of entrepreneurship and improve their self-efficacy and entrepreneurial attitudes. In this regard, Policymakers could play an important role in planting the seeds of entrepreneurship early on by providing the resources for students to launch small businesses during their student life. Policymakers could also use personal values as criteria to filter out potential entrepreneurs to better allocate national entrepreneurship resources.

### Limitations and Suggestions for Future Studies

Like any other research, the present study has some limitations. First, the data of the present study are self-reported, and they were collected using a questionnaire, which could be influenced by social desirability. Therefore, it is recommended that future studies try to collect data using multiple sources (such as interview and observation), and this may reduce the likelihood of skewed common method and social desirability. The second limitation is the cross-sectional nature of the present study, so the use of the SEM does not prove the causality. Although previous studies show that entrepreneurial intentions are the best predictor of entrepreneurial behavior, using longitudinal studies is recommended to researchers, and this could provide more opportunity for the investigation of causality. Moreover, research shows that there may be a gap between entrepreneurial intentions and entrepreneurial behavior (Kautonen et al., 2015). Therefore, the effect of personal values on the intention–behavior link requires further research in the future. Third, the questionnaire by Liñán and Chen (2009), which was used to measure subjective norms, is deemed suitable for use in samples of students but does not explicitly encompass Ajzen’s (2002) subjective norms definition. Future studies should try to replicate our findings using a questionnaire that explicitly follows the recommendations of Ajzen (2002). The fourth limitation is that, following previous studies (Karimi et al., 2017; Gorgievski et al., 2018; Karimi, 2019; Kruse et al., 2019), data were collected in the context of the university from students. They may have different values compared to other groups in society, so future studies should collect data from other groups, such as young entrepreneurs. Although there are other stimulating variables that could act as a mediator, in this study, just the antecedents of the TPB were used as the mediators of personal values. Future studies could examine whether or not there are more mediating variables to influence personal values on entrepreneurial intentions. Future research could also expand our study by testing the effects of collectivistic values on entrepreneurial intentions and behaviors. There is no agreement between scholars on whether individualism and collectivism are the opposite poles of the same dimension (Liñán et al., 2016). We have only focused on the individualistic values, but the consideration of collectivistic values may increase our understanding of the role of personal values in entrepreneurship.

## Conclusion

This article aimed to address a very important gap found in the cognitive entrepreneurship literature, namely the role of personal values in forming entrepreneurial intentions in developing countries, using the TPB (Ajzen, 1991) and Schwartz’s (1992) theory of personal values. Results indicate that openness to change and self-enhancement values are not directly related to entrepreneurial intentions. However, these personal values are indirectly related to entrepreneurial intentions through attitude toward entrepreneurship and perceived behavioral control. Furthermore, the results show that the relationships within the research model are not different between the two developing countries of Iran and Afghanistan. Thus, it attempted to respond to the call to reveal the role of personal values in entrepreneurship, particularly in developing countries, which has recently been emphasized in the entrepreneurship literature (Fayolle and Liñán, 2014; Liñán and Fayolle, 2015; Liñán et al., 2016). Other researchers are recommended to investigate whether our findings can be replicated in different cultural contexts and sample populations. As noted earlier, future research may also assess whether collectivistic personal values would have different effects on entrepreneurial intentions, its antecedents, and whether other psychological factors would mediate the relationships among these values and entrepreneurial intentions and behavior. In conclusion, this research provides evidence that individualistic personal values are important in determining entrepreneurial intentions and offer insights regarding the importance of these values in the entrepreneurial process in developing countries. Our results can also inform the development of promotional programs that foster students’ intentions to start a new business.

## Data Availability Statement

All datasets generated for this study are included in the article/supplementary material.

## Ethics Statement

Ethical review and approval were not required for the study on human participants in accordance with the local legislation and institutional requirements. The informed consent of the participants was implied through survey completion.

## Author Contributions

SK contributed to the design and implementation of the research, to the analysis of the results and to the writing of the manuscript. Both authors contributed to the article and approved the submitted version.

## Conflict of Interest

The authors declare that the research was conducted in the absence of any commercial or financial relationships that could be construed as a potential conflict of interest.

## References

[B1] Adam A. F. Fayolle A. (2015). Bridging the entrepreneurial intention-behaviour gap: the role of commitment and implementation intention. *Int. J. Entrepren. Small Bus.* 25 36–54. 10.1504/ijesb.2015.068775

[B2] Ajzen I. (1991). The theory of planned behavior. *Organ. Behav. Hum. Decis. Process.* 50 179–211.

[B3] Ajzen I. (2002). *Constructing a TPB Questionnaire: Conceptual and Methodological Considerations.* Avaliable at: www-unix. oit. umass. edu/-aizen (accessed January, 08th 2019).

[B4] Ajzen I. Fishbein M. (2005). “The influence of attitudes on behavior,”in *The Handbook of Attitudes*, eds Albarracín D. Johnson B. T. Zanna M. P. (New Jersey: Lawrence Erlbaum Associates Publishers), 173–221.

[B5] Azizi S. Jamali D. (2016). CSR in afghanistan: a global CSR agenda in areas of limited statehood. *South Asian J. Glob. Bus. Res.* 5 165–189. 10.1108/sajgbr-01-2015-0007

[B6] Bardi A. Lee J. A. Towfigh N. Soutar G. (2009). The structure of intra-individual value change. *J. Pers. Soc. Psychol.* 97 913–929. 19857010 10.1037/a0016617

[B7] Bardi A. Schwartz S. H. (2003). Values and behavior: strength and structure of relations. *Personal. Soc. Psychol. Bull.* 29 1207–1220. 10.1177/0146167203254602 15189583

[B8] Bernard M. M. Maio G. R. Olson J. M. (2003). The vulnerability of values to attack: inoculation of values and value-relevant attitudes. *Personal. Soc. Psychol. Bull.* 29 63–75. 10.1177/0146167202238372 15272960

[B9] Carree M. Thurik R. (2003). “The impact of entrepreneurship on economic growth,” in *Handbook of Entrepreneurship Research*, eds Audretsch D. B. Acs Z. J. (Boston, MA: Kluwer Academic Publishers), 437–471. 10.1007/0-387-24519-7_17

[B10] Charters S. Velikova N. Ritchie C. Fountain J. Thach L. Dodd T. H. (2011). Generation Y and sparkling wines: a cross-cultural perspective. *Int. J. Wine Bus. Res.* 23 161–175. 10.1108/17511061111143016

[B11] Cieciuch J. (2017). “Exploring the complicated relationship between values and behaviour,” in *Values and Behavior*, eds S. Roccas, and L. Sagiv (Cham: Springer), 237–247. 10.1007/978-3-319-56352-7_11

[B12] Engle R. L. Dimitriadi N. Gavidia J. V. Schlaegel C. Delanoe S. Alvarado I. (2010). Entrepreneurial intent: a twelve-country evaluation of Ajzen’s model of planned behavior. *Int. J. Entrepren. Behav. Res.* 16 35–57. 10.1108/13552551011020063

[B13] Espıritu-Olmos R. Sastre-Castillo M. A. (2015). Personality traits versus work values: comparing psychological theories on entrepreneurial intention. *J. Bus. Res.* 68 1595–1598. 10.1016/j.jbusres.2015.02.001

[B14] Fayolle A. Liñán F. (2014). The future of research on entrepreneurial intentions. *J. Bus. Res.* 67 663–666. 10.1016/j.jbusres.2013.11.024

[B15] Fishbein M. Ajzen I. (2011). *Predicting and Changing Behavior: The Reasoned Action Approach.* Hove: Psychology press.

[B16] Fitzsimmons J. R. Douglas E. J. (2011). Interaction between feasibility and desirability in the formation of entrepreneurial intentions. *J. Bus. Ventur.* 26 431–440. 10.1016/j.jbusvent.2010.01.001

[B17] Fornell C. Larcker D. F. (1981). Structural equation models with unobservable variables and measurement error: algebra and statistics. *J. Market. Res.* 18 382–388. 10.2307/3150980

[B18] Goodnow J. J. (1997). “Parenting and the “transmission” and “internalization” of values: from social-cultural perspectives to within-family analyses,” in *Handbook of Parenting and the Transmission of Values*, eds Grusec J. E. Kuczynski L. (New York, NY: Wiley), 333–361.

[B19] Gorgievski M. J. Stephan U. Laguna M. Moriano J. A. (2018). Predicting entrepreneurial career intentions: values and the theory of planned behavior. *J. Career Assess.* 26 457–475. 10.1177/1069072717714541 30443149 PMC6196350

[B20] Griffiths M. D. Kickul J. R. Carsrud A. L. (2009). Government bureaucracy, transactional impediments, and entrepreneurial intentions. *Int. Small Bus. J.* 27 626–645. 10.1177/0266242609338752

[B21] Hair J. F. Hult G. T. M. Ringle C. M. Sarstedt M. (2014). *A Primer on Partial Least Squares Structural Equation Modeling (PLS-SEM).* Thousand Oaks, CA: Sage.

[B22] Hair J. F. Jr. Sarstedt M. Ringle C. M. Gudergan S. P. (2017). *Advanced Issues in Partial Least Squares Structural Equation Modeling.* Thousand Oaks, CA: Sage Publications.

[B23] Hambleton R. K. (1994). Guidelines for adapting educational and psychological tests: a progress report. *Eur. J. Psychol. Assess.* 10 229–244.

[B24] Holland D. V. Shepherd D. A. (2013). Deciding to persist: adversity, values, and entrepreneurs’ decision policies. *Entrepren. Theory Pract.* 37 331–358. 10.1111/j.1540-6520.2011.00468.x

[B25] Homer P. M. Kahle L. R. (1988). A structural equation test of the value-attitude-behavior hierarchy. *J. Pers. Soc. Psychol.* 54 638–648. 10.1037/0022-3514.54.4.638

[B26] Jaén I. Liñán F. (2013). Work values in a changing economic environment: the role of entrepreneurial capital. *Int. J. Manpow.* 34 939–960. 10.1108/ijm-07-2013-0166

[B27] Karimi S. (2019). The role of entrepreneurial passion in the formation of students’ entrepreneurial intentions. *Appl. Econ.* 52 331–344. 10.1080/00036846.2019.1645287

[B28] Karimi S. Biemans H. J. Naderi Mahdei K. Lans T. Chizari M. Mulder M. (2017). Testing the relationship between personality characteristics, contextual factors and entrepreneurial intentions in a developing country. *Int. J. Psychol.* 52 227–240. 10.1002/ijop.12209 26334129

[B29] Karimi S. Biemans H. J. A. Lans T. Chizari M. Mulder M. (2014). Effects of role models and gender on students’ entrepreneurial intentions. *Eur. J. Train. Dev.* 38 694–727. 10.1108/ejtd-03-2013-0036

[B30] Karimi S. Biemans H. J. Lans T. Mulder M. (2019). Understanding the role of cultural orientations in the formation of entrepreneurial intentions in Iran. *J. Career Dev.* 10.1177/0894845319880264 [Epub ahead of print].

[B31] Kautonen T. van Gelderen M. Fink M. (2015). Robustness of the theory of planned behavior in predicting entrepreneurial intentions and actions. *Entrepren. Theory Pract.* 39 655–674. 10.1111/etap.12056

[B32] Kessler A. Frank H. (2009). Nascent entrepreneurship in a longitudinal perspective: the impact of person, environment, resources and the founding process on the decision to start business activities. *Int. Small Bus. J.* 27 720–742. 10.1177/0266242609344363

[B33] Knafo A. Sagiv L. (2004). Values and work environment: mapping 32 occupations. *Eur. J. Psychol. Educ.* 19 255–273. 10.1007/bf03173223

[B34] Kolvereid L. Isaksen E. (2006). New business start-up and subsequent entry into self-employment. *J. Bus. Ventur.* 21 866–885. 10.1016/j.jbusvent.2005.06.008

[B35] Konsky C. Eguchi M. Blue J. Kapoor S. (2000). Individualist-collectivist values: American, Indian and Japanese cross-cultural study. *Intercult. Commun. Stud.* 9 69–84.

[B36] Krueger N. F. Jr. (2007). What lies beneath? The experiential essence of entrepreneurial thinking. *Entrepren. Theory Pract.* 31 123–138. 10.1111/j.1540-6520.2007.00166.x

[B37] Krueger N. F. (2009). “Entrepreneurial intentions are dead: long live entrepreneurial intentions,” in *Revisiting the Entrepreneurial Mind*, eds A. L. Carsrud, and M. Brännback (Cham: Springer), 13–34. 10.1007/978-3-319-45544-0_2

[B38] Kruse P. Wach D. Costa S. Moriano J. A. (2019). Values matter, Don’t They?–combining theory of planned behavior and personal values as predictors of social entrepreneurial intention. *J. Soc. Entrepren.* 10 55–83. 10.1080/19420676.2018.1541003

[B39] Levasseur L. Tang J. Karami M. Busenitz L. Kacmar K. M. (2020). Increasing alertness to new opportunities: the influence of positive affect and implications for innovation. *Asia Pacific J. Manag.* 1–23 10.1007/s10490-020-09724-y [Epub ahead of print].

[B40] Liñán F. Chen Y. W. (2009). Development and cross–cultural application of a specific instrument to measure entrepreneurial intentions. *Entrepren. Theory Pract.* 33 593–617. 10.1111/j.1540-6520.2009.00318.x

[B41] Liñán F. Fayolle A. (2015). A systematic literature review on entrepreneurial intentions: citation, thematic analyses, and research agenda. *Int. Entrepren. Manag. J.* 11 907–933. 10.1007/s11365-015-0356-5

[B42] Liñán F. Kurczewska A. (2017). “Why are some individuals willing to pursue opportunities and others aren’t? The role of individual values,” in *Research Handbook on Entrepreneurial Opportunities: Reopening the Debate*, eds Eger-Jarniou C. L. Tegtmeier S. (Cheltenham: Edward Elgar), 263–284. 10.4337/9781783475445.00019

[B43] Liñán F. Moriano J. A. Jaén I. (2016). Individualism and entrepreneurship: does the pattern depend on the social context? *Int. Small Bus. J.* 34 760–776. 10.1177/0266242615584646

[B44] Lüthje C. Franke N. (2003). The ‘making’of an entrepreneur: testing a model of entrepreneurial intent among engineering students at MIT. *R D Manag.* 33 135–147. 10.1111/1467-9310.00288

[B45] Looi K. H. (2019). Undergraduates’ motivations for entrepreneurial intentions: the role of individualistic values and ethnicity. *J. Educ. Work* 32 465–483. 10.1080/13639080.2019.1640866

[B46] MacBride E. (2016). *Seven Reasons Iran could Become an Entrepreneurial Powerhouse.* Avaliable at: https://www.forbes.com/sites/elizabethmacbride/2016/04/30/seven-reasons-iran-is-likely-to-be-an-entrepreneurialpowerhouse/#3acc07f7ec8c (accessed 8 September 2019).

[B47] Maio G. R. (2010). “Mental representations of social values,”in *Advances in Experimental social Psychology*, Vol. 42 ed. M. P. Zanna (Cambridge, MA: Academic Press), 1–43. 10.1016/s0065-2601(10)42001-8

[B48] Morales C. Holtschlag C. Masuda A. D. Marquina P. (2019). In which cultural contexts do individual values explain entrepreneurship? An integrative values framework using Schwartz’s theories. *Int. Small Bus. J.* 37 241–267. 10.1177/0266242618811890

[B49] Morales C. E. Holtschlag C. (2013). Post materialist values and entrepreneurship: a multilevel approach. *Int. J. Entrepren. Behav. Res.* 19 266–282. 10.1108/13552551311330174

[B50] Moriano J. A. Gorgievski M. Laguna M. Stephan U. Zarafshani K. (2012). A cross-cultural approach to understanding entrepreneurial intention. *J. Career Dev.* 39 162–185. 10.1177/0894845310384481

[B51] Moriano J. A. Palací F. J. Morales J. F. (2007). The psychosocial profile of the university entrepreneur. *Psychol. Spain* 11 72–84.

[B52] Muhammad A. Akbar S. Dalziel M. (2011). The journey to develop educated entrepreneurs: prospects and problems of Afghan businessmen. *Educ. Train.* 53 433–447. 10.1108/00400911111147730

[B53] Noseleit F. (2010). “The entrepreneurial culture: guiding principles of the self-employed,” in *Entrepreneurship and Culture*, eds Freytag A. Thurik R. (Berlin: Springer-Verlag), 41–54. 10.1007/978-3-540-87910-7_3

[B54] O’Neill J. Wilson D. Purushothaman R. Stupnytska A. (2005). Global economics paper no:134-How solid are the BRICs. *Accessed* 8:2017.

[B55] Preacher K. J. Hayes A. F. (2004). SPSS and SAS procedures for estimating indirect effects in simple mediation models. *Behav. Res. Methods Instrume. Comput.* 36 717–731. 10.3758/bf03206553 15641418

[B56] Preacher K. J. Hayes A. F. (2008). Asymptotic and resampling strategies for assessing and comparing indirect effects in multiple mediator models. *Behav. Res. Methods* 40 879–891. 10.3758/brm.40.3.879 18697684

[B57] Ringle C. M. Wende S. Becker J. (2017). *SmartPLS—Statistical Software for Structural Equation Modeling.* Boenningstedt: SmartPLS GmbH Accessed: Jan. 15, 2020. [Online]. Available: http://www.smartpls.com.

[B58] Rohan M. J. (2000). A rose by any name? The values construct. *Pers. Soc. Psychol. Rev.* 4 255–277. 10.1207/s15327957pspr0403_4

[B59] Rokeach M. (1973). *The Nature of Human Values.* New York, NK: Free press.

[B60] Sagiv L. (2002). Vocational interests and basic values. *J. Career Assess.* 10 233–257. 10.1177/1069072702010002007

[B61] Schwartz S. H. (1992). “Universals in the content and structure of values: theory and empirical tests in 20 countries,” in *Advances in Experimental Social Psychology*, ed. Zanna M. (New York, NY: Academic Press), 1–65. 10.1016/s0065-2601(08)60281-6

[B62] Schwartz S. H. (1994). Are there universal aspects in the structure and contents of human values? *J. Soc. Issues* 50 19–45. 10.1111/j.1540-4560.1994.tb01196.x

[B63] Schwartz S. H. (2006). A theory of cultural value orientations: explications and applications. *Comp. Sociol.* 5 137–182. 10.1163/156913306778667357

[B64] Schwartz S. H. (2011). Studying values: personal adventure. Future Directions. *J. Cross Cult. Psychol.* 42 307–319. 10.1177/0022022110396925

[B65] Schwartz S. H. Boehnke K. (2004). Evaluating the structure of human values with confirmatory factor analysis. *J. Res. Personal.* 38 230–255. 10.1016/s0092-6566(03)00069-2

[B66] Schwartz S. H. (2015). “Basic individual values: sources and consequences,” in *Handbook of Value: Perspectives from Economics, Neuroscience, Philosophy, Psychology and Sociology*, eds Sander D. Brosch T. (Oxford: Oxford University Press), 63–84.

[B67] Schwartz S. H. Melech G. Lehmann A. Burgess S. Harris M. Owens V. (2001). Extending the cross-cultural validity of the theory of basic human values with a different method of measurement. *J. Cross Cult. Psychol.* 32 519–542. 10.1177/0022022101032005001

[B68] Shirokova G. Osiyevskyy O. Bogatyreva K. (2016). Exploring the intention–behavior link in student entrepreneurship: moderating effects of individual and environmental characteristics. *Eur. Manag. J.* 34 386–399. 10.1016/j.emj.2015.12.007

[B69] Tomczyk D. Lee J. Winslow E. (2013). Entrepreneurs’ personal values, compensation, and high growth firm performance. *J. Small Bus. Manag.* 51 66–82. 10.1111/j.1540-627x.2012.00374.x

[B70] van Stel A. Carree M. Thurik R. (2005). The effect of entrepreneurial activity on national economic growth. *Small Bus. Econ.* 24 311–321. 10.1007/s11187-005-1996-6

